# Surgical and local control outcomes after sequential short-course radiation therapy and chemotherapy for rectal cancer

**DOI:** 10.1016/j.sopen.2024.01.015

**Published:** 2024-01-23

**Authors:** I-Chia Liu, Susan Gearhart, Suqi Ke, Chen Hu, Haniee Chung, Jonathan Efron, Alodia Gabre-Kidan, Peter Najjar, Chady Atallah, Bashar Safar, Eric S. Christenson, Nilofer S. Azad, Valerie Lee, Atif Zaheer, Jacqueline E. Birkness-Gartman, Abhinav V. Reddy, Amol K. Narang, Jeffrey Meyer

**Affiliations:** a Department of Radiation Oncology & Molecular Radiation Sciences, Johns Hopkins University School of Medicine, Baltimore, MD, USA; b Department of Surgery, Colorectal Research Unit, Ravitch Division of Colorectal Surgery, Johns Hopkins University School of Medicine, Baltimore, MD, USA; c Division of Biostatistics and Bioinformatics, Sidney Kimmel Comprehensive Cancer Center, Johns Hopkins University School of Medicine, Baltimore, MD, USA; d Department of Surgery, Division of Colon and Rectal Surgery, NYU Langone Health, New York, NY, USA; e Department of Oncology, Sidney Kimmel Comprehensive Cancer Center at Johns Hopkins, Baltimore, MD, USA; f Department of Radiology and Radiological Science, Johns Hopkins University School of Medicine, Baltimore, MD, USA; g Department of Pathology, Johns Hopkins University School of Medicine, Baltimore, MD, USA

**Keywords:** Rectal cancer, Radiation therapy, Proctectomy

## Abstract

**Background:**

Total neoadjuvant therapy (TNT) is an accepted approach for the management of locally advanced rectal cancer (LARC) and is associated with a decreased risk of development of metastatic disease compared to standard neoadjuvant therapy. However, questions remain regarding surgical outcomes and local control in patients who proceed to surgery, particularly when radiation is given first in the neoadjuvant sequence. We report on our institution's experience with patients who underwent short-course radiation therapy, consolidation chemotherapy, and surgery.

**Methods:**

We retrospectively reviewed surgical specimen outcomes, postoperative complications, and local/pelvic control in a large cohort of patients with LARC who underwent neoadjuvant therapy incorporating upfront short-course radiation therapy followed by consolidation chemotherapy.

**Results:**

In our cohort of 83 patients who proceeded to surgery, a complete/near-complete mesorectal specimen was achieved in 90 % of patients. This outcome was not associated with the time interval from completion of radiation to surgery. Postoperative complications were acceptably low. Local control at two years was 93.4 % for all patients- 97.6 % for those with low-risk disease and 90.4 % for high-risk disease.

**Conclusion:**

Upfront short-course radiation therapy and consolidation chemotherapy is an effective treatment course. Extended interval from completion of short-course radiation therapy did not impact surgical specimen quality.

## Introduction

The majority of tumor recurrences following primary rectal cancer management with modern neoadjuvant therapy and total mesorectal excision (TME) involve extra-pelvic metastatic disease, not local relapse [1]. The value of adjuvant chemotherapy in reducing these distant failures is controversial and not robustly supported by results from randomized clinical trials [2]. Total neoadjuvant therapy (TNT) is a treatment approach designed to address the problem of distant disease control, incorporating early, pre-surgical, sequencing of systemic therapy [3]. Evidence from recent randomized trials investigating the TNT concept in locally advanced rectal cancer (LARC) management- RAPIDO and PRODIGE 23- provided support for its use, as both trials demonstrated a reduced rate of metastatic disease development relative to neoadjuvant treatment with chemoradiotherapy [4,5]. The STELLAR trial demonstrated improved overall survival in TNT-treated patients compared to chemoradiotherapy, although metastasis-free survival rates were similar between the two arms [6]. TNT may also allow for a greater likelihood of complete local disease eradication, leading to higher rates of organ preservation/non-operative management (NOM) compared to traditional chemoradiotherapy or short-course radiation therapy alone [[7], [8], [9]].

Robust biomarkers to aid in determining which patients are most likely to benefit from TNT are lacking, however, and many patients will have local residual disease following the completion of neoadjuvant therapy. In the radiation-first approach to TNT (typically involving short-course radiation therapy or long-course chemoradiotherapy followed by consolidation chemotherapy), there is a significantly extended interval from the completion of radiation therapy to surgery. Extended delay to surgery may impact the technical difficulty of the TME dissection and the resulting quality of the surgical specimen, as well as influence the risk profile of postoperative complications. TME completeness is an important factor in reducing local-regional failures, as demonstrated in the pre-TNT era [10]. In the RAPIDO trial, where short-course radiation therapy was delivered prior to consolidation chemotherapy in the TNT arm, the TME specimen was more likely to be disrupted in the TNT-treated patients, possibly a reflection of the lengthy interval from radiation to surgery (median of 23.6 weeks versus median of 8.9 weeks in the chemoradiotherapy arm) [4]. In a recent update of results from RAPIDO, although the beneficial impact of TNT on distant disease failure was maintained, there was now a statistically significant increase in local-regional tumor recurrence in patients undergoing R0 or R1 resection in the TNT arm compared to patients treated with standard chemoradiotherapy followed by surgery [11]. Other studies have also demonstrated an association between prolonged interval from completion of radiation to surgery with suboptimal surgical specimen outcomes [12,13] Thus, it is imperative to understand if continued use of the TNT platform will be at the cost of an increased risk of local failure, even if modest, and to understand what clinical factors may predict for this risk.

At our institution, we have been using extended neoadjuvant therapy using a radiation-first approach- incorporating short-course radiation therapy (SCRT)- for over 5 years for treatment of LARC. We previously reported our early results with this approach, as well as our experience with nonoperative management for patients with LARC following complete response to SCRT and consolidation chemotherapy [9,14]. In recent years we have increased the duration of the consolidation component to the true TNT approach used in RAPIDO (i.e., moved from near-TNT to TNT). In this report we update our experience to determine the impact of extended neoadjuvant therapy on surgical specimen quality and postoperative complications in the cohort who proceeded to surgery and also review intermediate-term local control outcomes.

## Materials and methods

### Study design

This study reflects the retrospective results of a single-institution experience and was approved by our institutional review board (IRB00194005, approval date 12/10/2018). We retrospectively collected data on baseline characteristics, treatment details, and outcomes of patients with LARC (stage II or III per AJCC guidelines) who were treated with SCRT followed by consolidation chemotherapy (extended neoadjuvant therapy) from April 2017 to May 2023. Routine staging involved MRI of the pelvis for local-regional tumor staging and CT imaging of the chest, abdomen, and pelvis to exclude distant disease.

Patients were divided into two main groups. The first group, the primary focus of this study, consisted of patients who underwent neoadjuvant treatment followed by surgery (NTx/Surgery group). The second group consisted of those who proceeded through neoadjuvant therapy, achieved a complete or near-complete clinical response, and deferred surgery to be followed on a nonoperative management pathway (NOM group). If local regrowth of disease was detected, patients were considered for salvage surgery.

High-risk clinical features were based on MRI staging and included the following: T4a or T4b disease, involved circumferential resection margin as assessed by MRI, extramural vascular invasion, and/or extramesorectal/lateral (internal iliac and/or pelvic sidewall) lymphadenopathy. High-risk cancer presentations were defined as having one or more of these features, and low-risk cancer presentations were defined as having none of these features.

### Treatment

Details of the treatment course have been previously described but are summarized here [9,14]. Patients underwent CT simulation for radiation therapy planning. The clinical target volume consisted of the gross tumor volume and at-risk regional lymph node volumes: mesorectal, presacral, internal iliac, and pelvic sidewall. Patients were treated with either 3D conformal or intensity-modulated radiation therapy. The SCRT prescription dose was 25 Gy in 5 fractions (5 Gy × 5 fractions) prescribed to target coverage. Radiation was delivered on consecutive weekdays and typically involved image guidance with cone-beam CT.

Consolidation chemotherapy began approximately 2–3 weeks after the completion of the radiation course. Patients received either 5-FU/leucovorin/oxaliplatin (mFOLFOX) or capecitabine/oxaliplatin (CAPOX). FOLFOX was repeated cyclically every 2 weeks (defined as one dose) and CAPOX was repeated every 3 weeks (defined as one dose). The treating medical oncologist evaluated the need for dose reduction and postponement of cycles for patients on a case-by-case basis.

Upon completion of chemotherapy, patients underwent reassessment for surgery with examination, repeat endoscopy, and imaging in most cases. Patients achieving a complete response (complete on endoscopic and digital exam evaluation) after neoadjuvant therapy were advised on the risks and benefits of a nonoperative management course; patients who elected this were followed with endoscopic and imaging surveillance, and constitute the NOM group [9,15]. The recommendation for an incomplete response following neoadjuvant therapy was surgery. These patients and those electing surgery constitute the NTx/Surgery group. The operating surgeon determined their approach to proctectomy. All surgeries employed the principles of total mesorectal excision (TME).

### Outcomes

For analysis of proctectomy outcomes, we evaluated patients in the NTx/Surgery group. We collected data on the completeness of the TME specimen, pathologic T and N stage, and margin status (including radial and distal) from the specimen pathology report. Quality of the mesorectum specimen was assessed per College of American Pathologists standards. For the outcome of surgical margin assessment, negative margins for the specimen were defined as uninvolved distal margins (of any distance), and radial margins of >1 mm. We defined optimal surgical outcome as those cases with complete or near-complete TME specimen as well as distal and radial margins of >1 mm, following the ACOSOG Z6501 convention [16]. We also recorded the length of hospital stay and postoperative adverse events for up to 30 days following surgery. Postoperative complication severity was graded per the Clavien-Dindo classification, with grade 3 or higher complications considered high-grade [17]. Only the highest morbidity event was counted per patient.

Patients in both the NTx/Surgery and NOM groups were pooled for analysis of local control. Local failure was defined as regrowth of tumor in the pelvis, at the anastomotic site, presacral site, and/or pelvic sidewalls, and was assessed by review of postoperative surveillance follow-up imaging of the pelvis. Biopsy was not required for definitive proof of local failure if imaging was consistent with recurrence, although follow-up assessment (biopsy or subsequent imaging) was needed for equivocal findings to declare local recurrence. Local recurrence for patients initially followed in the NOM group (“first” local failure) was not considered an event for local control analysis (these patients typically underwent salvage surgery), but local failure subsequent to this (“second” local failure) was considered an event. Time to failure was calculated from the time of surgery for patients in the NTx/Surgery group, and from the time of completion of radiation therapy for patients in the NOM group. Patients who had surgery were censored at the time of last pelvic imaging (CT or MRI) if there was no evidence of local recurrence. Patients in the NOM group and never had salvage surgery were censored at the time of last endoscopic follow-up. Local control was assessed through June 2023.

### Statistical analysis

Descriptive statistics are presented for baseline patient characteristics, disease status, and treatment duration and type as median values with range, mean with standard deviation, and percentages. The Wilcoxon and Fisher's exact tests were used to compare group differences for continuous and categorical variables, respectively. Univariate logistic regression was performed on clinically relevant variables for the main endpoints of TME quality (complete/near-complete versus incomplete) and high-grade postoperative morbidity (Clavien-Dindo grade ≥ 3 versus lower). Multivariable logistic regression was carried out on variables of high interest from univariate modeling, to control for potential confounding. The functional form of continuous variables in the aforementioned regression models, such as age and time interval from completion of radiation therapy to surgery, was explored using cubic splines. Accordingly, we determined a non-linear effect of the days from completion of radiation therapy to surgery variable for the endpoint of high-grade postoperative morbidity, and thus used a piecewise linear model with the cut point of 180 days to surgery to capture such a U-shape effect in this particular analysis. Local control was assessed using the Kaplan-Meier method and the log-rank test.

## Results

### Overview of institutional experience with neoadjuvant short-course radiation therapy and consolidation chemotherapy

Over the reviewed time span of the study, 151 patients with LARC were treated with SCRT and consolidation chemotherapy. Twenty-six patients remained on treatment and were not further analyzed in this study. Of the 125 patients who had completed their neoadjuvant course, 41 (33 %) proceeded to nonoperative management (NOM group) and 83 (67 %) proceeded to surgery (NTx/Surgery group). One patient changed treatment to immunotherapy and did not proceed to surgery and was not included in either the NOM or NTx/Surgery cohorts, and was not further analyzed.

**Fig. 1:**
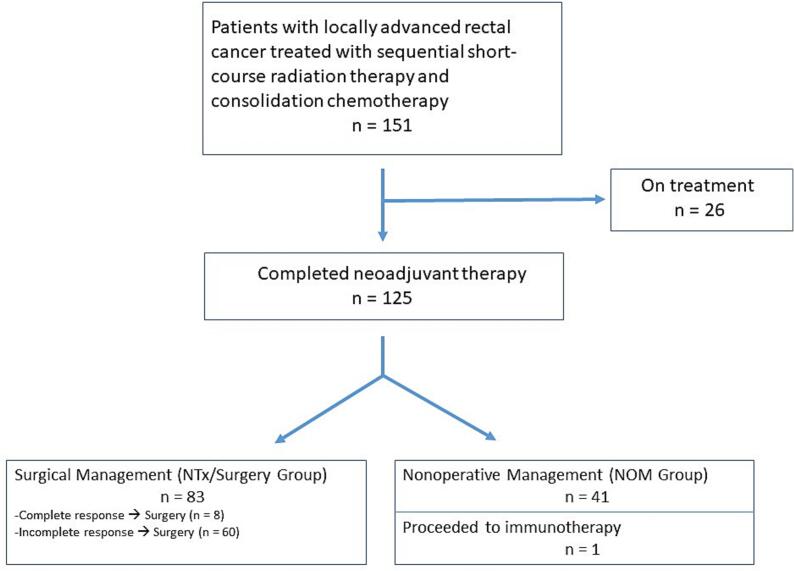
Treatment pathway at our institution.

In the NOM group, 23 patients (56 %) had high-risk presentations. Of the 83 patients in the NTx/Surgery group, 53 (64 %) had high-risk presentations. Forty-four patients (53 %) in the NTx/Surgery group received 12 or more weeks of consolidation chemotherapy. Fig. 1 shows the path of the entire cohort through neoadjuvant therapy and Table 1 shows details of the patients in the NTx/Surgery group who served as the basis for subsequent analyses of postoperative outcomes. Practice patterns changed over the time reviewed in this study, with an increase in the amount of consolidation chemotherapy seen in the latter years. Forty-three patients (51.8 %) received postoperative chemotherapy; our practice adopted true total neoadjuvant therapy only in the later years. Of note, the median time from completion of radiation therapy to surgery was 22.9 weeks, similar to the 23.6 week interval reported in RAPIDO.

**Table 1:** Clinical and treatments details of NTx/surgery patient cohort.

Number of patients	83
Median age at diagnosis (range)	54 years (31–78)
Body mass index (mean ± SD)	27.3 ± 4.5
Sex	
Female	27 (32.5 %)
Male	56 (67.5 %)
Tumor location - distance (cm) from anal verge	
Low (0–5)	36 (43.4 %)
Mid (> 5–10)	35 (42.2 %)
High (> 10–15)	12 (14.5 %)
Clinical stage	
Stage II	10 (12 %)
Stage III	73 (88 %)
Neoadjuvant chemotherapy type and number of doses	
mFOLFOX	55 (66.3 %)
Median number of doses (range)	4 (3–12)
CAPOX	26 (31.3 %)
Median number of doses (range)	6 (2–6)
CAPOX then mFOLFOX	2 (2.4 %)
One patient received 1 dose of CAPOX followed by 5 doses of mFOLFOX. The other patient received 3 doses of CAPOX followed by 4 doses of mFOLFOX	
Median time from completion of radiation therapy to surgery (range)	160 days (86–326)
Type of surgery	
Low anterior resection (LAR)	68 (81.9 %)
Total number of abdominoperineal resection (APR)	15 (18.1 %)
Surgical approach	
Open	23 (27.71 %)
Laparoscopic	8 (9.64 %)
Robotic	52 (62.65 %)

### Proctectomy approach and histopathologic outcomes

Sixty-eight patients in the NTx/Surgery group (82 %) underwent low anterior resection, and most surgeries (62 %) were performed with robotic-assisted laparoscopy (Table 1). A complete histopathologic response to neoadjuvant therapy was seen in 21 (25 %) surgical specimens. One patient with residual high-grade dysplasia, but no residual invasive cancer, in the specimen was included in the pathologic complete response group calculation and in the statistical analyses. The pathologic complete response rate was 24 % if this patient is not included in the pathologic complete response group.

### Proctectomy outcomes: TME specimen quality and margins

Of the 83 surgeries in the NTx/Surgery group, 75 (90 %) yielded a complete or near-complete TME specimen, as assessed by the pathologist. Negative surgical margins were obtained in 77 surgeries (93 %). Defining optimal surgical outcome as a composite of complete/near-complete TME specimen and margins of >1 mm, 67 surgeries (81 %) reached this standard [16]. Table 2 shows summary statistics of the patients with respect to complete/near-complete versus incomplete TME specimen status, and Table 3 shows results of univariate and multivariable analysis for predictors of poor-quality TME outcome. In univariate analysis, duration of chemotherapy (< 12 weeks or ≥ 12 weeks), interval from completion of radiation to surgery modeled as a continuous variable, and type of surgery were of borderline significance. In multivariable analysis, duration of chemotherapy and interval from completion of radiation therapy to surgery were modeled separately because of collinearity (Table 3). Neither the interval duration nor type of surgery were significantly associated with an increased risk of incomplete TME specimen.

**Table 2:** Comparison of patients with and without complete mesorectal surgical specimens in the NTx/surgery cohort.

	Incomplete mesorectum (*N* = 8)	Complete/near-complete mesorectum (*N* = 75)	*P* value
Age (years)			
Mean (SD)	56.6 (11.6)	55.9 (12.4)	0.951
Median (range)	55 (43–71)	54 (31–78)	
Sex			
Female	3 (37.5 %)	24 (32.0 %)	0.711
Male	5 (62.5 %)	51 (68.0 %)	
Stage of disease			
II	0 (0 %)	10 (13.3 %)	0.587
III	8 (100 %)	65 (86.7 %)	
Duration of chemotherapy			
<12 weeks	1 (12.5 %)	38 (50.7 %)	0.0611
≥12 weeks	7 (87.5 %)	37 (49.3 %)	
Time from completion of radiation therapy to surgery (days)			
Mean (SD)	191 (62.9)	155 (47.9)	0.127
Median (range)	182 (127–326)	151 (86–273)	
Type of surgery			
LAR	5 (62.5 %)	63 (84.0 %)	0.153
APR	3 (37.5 %)	12 (16.0 %)	
Surgical approach			
Open	4 (50 %)	19 (25.3 %)	0.349
Laparoscopic	0 (0 %)	8 (10.7 %)	
Robotic	4 (50.0 %)	48 (64.0 %)	
Tumor location			
Low	4 (50 %)	32 (42.7 %)	0.361
Mid	2 (25.0 %)	33 (44.0 %)	
High	2 (25.0 %)	10 (13.3 %)	
BMI			
Mean (SD)	26.9 (4.48)	27.4 (4.51)	0.605
Pathologic response			
Incomplete	7 (87.5 %)	55 (73.3 %)	0.673
Complete	1 (12.5 %)	20 (26.7 %)

**Table 3:** Odds ratios for complete/near-complete mesorectal excision related to patient, cancer, and treatment features in the NTx/surgery cohort.

Covariate	Number of patients	Number of events^a^	Univariate analysis		Multivariable analysis	
			OR (95 % CI)	P value	OR (95 % CI)	P value
Age			1.00 (0.94–1.06)	0.877		
Sex						
Female	27	3	1 [reference]			
Male	56	5	1.27 (0.28–5.78)	0.753		
BMI			1.02 (0.87–1.21)	0.789		
Duration of chemotherapy						
<12 weeks	39	1	1 [reference]			
≥12 weeks	44	7	0.14 (0.02–1.19)	0.071		
Time from completion of radiation therapy to surgery (days)			0.99 (0.97–1.00)	0.069	0.99 (0.97–1.00)	0.134
Type of surgery						
LAR	68	5	1 [reference]			
APR	15	3	0.32 (0.07–1.51)	0.149	0.44 (0.08–2.37)	0.343
Tumor location						
Low	36	4	1 [reference]			
Mid	35	2	2.06 (0.35–12.06)	0.422		
High	12	2	0.63 (0.10–3.94)	0.617		
Pathologic response						
Incomplete	62	7	1 [reference]			
Complete	21	1	2.55 (0.29–22.0)	0.396		

a An event was defined as an incomplete mesorectal specimen.

### Postoperative morbidity

High-grade postoperative morbidity (up to 30 days following surgery) using the Clavien-Dindo classification was seen in 15 patients (18 %) as shown in Table 4. The 30-day readmission rate was 16 %. There were no deaths within 30 days of surgery. Univariate analysis showed that the type of surgery (APR vs LAR) was associated with an increased risk of high-grade postoperative morbidity, and this relationship was maintained in multivariable analysis (Table 5). Interval from completion of radiation therapy to surgery showed a complex relationship with high-grade morbidity, and was modeled as a continuous variable over two time periods- <180 days to surgery and ≥180 days to surgery. Interval was significantly inversely associated with high-grade morbidity in the <180 days to surgery group but not in the ≥180 days group.

**Table 4:** Comparison between patients with and without high-grade postoperative morbidity in the NTx/surgery cohort.

	Clavien-Dindo grade ≥ 3 (*N* = 15)	Clavien-Dindo grade < 3 (*N* = 68)	P value
Age (years)			
Mean (SD)	51.5 (11.8)	57.0 (12.3)	0.117
Median (range)	51 (34–71)	55.5 (31–78)	
Sex			
Female	3 (20 %)	24 (35.3 %)	0.365
Male	12 (80 %)	44 (64.7 %)	
Stage of disease			
II	0 (0 %)	10 (14.7 %)	0.196
III	15 (100 %)	58 (85.3 %)	
Duration of chemotherapy			
<12 weeks	10 (66.7 %)	29 (42.6 %)	0.152
≥12 weeks	3 (33.3 %)	39 (57.4 %)	
Time from completion of radiation therapy to surgery (days)			
Mean (SD)	143 (65.4)	162 (46.0)	0.0666
Median (range)	111 (95–326)	164 (86–273)	
Type of surgery			
LAR	13 (86.7 %)	59 (86.8 %)	0.0247*
APR	2 (13.3 %)	9 (13.2 %)	
Surgical approach			
Open	4 (26.7 %)	19 (27.9 %)	0.826
Laparoscopic	2 (13.3 %)	6 (8.8 %)	
Robotic	9 (60.0 %)	43 (63.2 %)	
Tumor location			
Low	9 (60.0 %)	27 (39.7 %)	0.453
Mid	5 (33.3 %)	30 (44.1 %)	
High	1 (6.7 %)	11 (16.2 %)	
BMI			
Mean (SD)	26.3 (3.80)	27.5 (4.61)	0.438

**Table 5:** Odds ratios for high-grade postoperative morbidity related to patient, cancer, and treatment features in the NTx/surgery cohort.

Covariate	Number of patients	Number of events^a^	Univariate analysis		Multivariable analysis	
			OR (95 % CI)	P value	OR (95 % CI)	P value
Age			0.96 (0.92–1.01)	0.125	0.95 (0.89–1.01)	0.077
Sex						
Female	27	3	1 [reference]			
Male	56	12	2.18 (0.56–8.50)	0.261		
BMI			0.94 (0.82–1.07)	0.343		
Duration of chemotherapy						
<12 weeks	39	10	1 [reference]			
≥12 weeks	44	5	0.37 (0.11–1.21)	0.099		
Interval from completion of radiation to surgery, per day, up to 180 days			0.99 (0.98–1.00)	0.184	0.97 (0.95–0.99)	0.014^a^
Interval from completion of radiation to surgery, per day, beyond 180 days			1.00 (0.98–1.03)	0.814	1.01 (0.98–1.04)	0.560
Type of surgery						
LAR	68	9	1 [reference]			
APR	15	6	4.37 (0.07–1.51)	0.021^a^	10.78 (1.84–63.03)	0.008^a^
Tumor location						
Low	36	9	1 [reference]			
Mid	35	5	0.50 (0.15–1.68)	0.262		
High	12	1	0.27 (0.03–2.42)	0.243		
Surgical approach						
Open	23	4	1 [reference]			
Laparoscopic	8	2	1.58 (0.23–10.90)	0.641		
Robotic	52	9	0.99 (0.27–3.63)	0.993		

a Event defined as ≥Grade 3 Clavien-Dindo morbidity.

### Local control

**Fig. 2:**
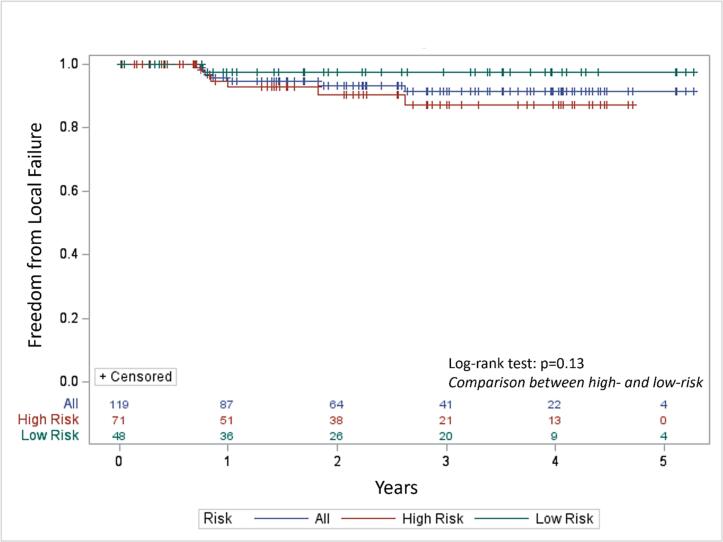
Freedom from local failure Kaplan-Meier curve.

For assessment of local control, we combined the NTx/Surgery and NOM cohorts. Three patients in the NTx/Surgery group were not assessable for local control because of lack of long-term imaging follow-up and were excluded from further analysis. In the NOM group, 9 patients developed local regrowth of disease (“first” failure). Seven of these patients underwent salvage surgery, with one subsequently developing a “second” local recurrence. Two patients with first failure did not undergo salvage surgery due to synchronous distant failure, and were excluded from analysis. Thus, 80 patients in the NTx/Surgery cohort and 39 in the NOM cohort were eligible for local control analysis. At a median follow-up of 2.1 years, 7 patients experienced local relapse of disease in the pelvis as detected on surveillance imaging (Fig. 2)- 6 in the NTx/Surgery group and the aforementioned one patient in the NOM group. Two-year local control was 93.4 % for all patients. Two-year local control was 97.6 % and 90.4 % for the low-risk and high-risk presentations, respectively (*p* = 0.13 by log-rank test).

## Discussion

Traditional arguments for neoadjuvant chemotherapy for solid tumors planned for definitive local therapy include improved patient compliance with treatment and earlier treatment of micrometastatic disease. The results from RAPDIO and PRODIGE 23 indeed showed a decrease in the rate of metastatic disease development in patients treated with TNT relative to neoadjuvant chemoradiotherapy, without clear impact on overall survival in the first few years of follow-up [4,5]. Pathologic complete response rates were also improved relative to chemoradiotherapy, and multiple studies have shown the potential for non-operative management following complete response to intensive TNT regimens [[4], [5], [6], [7], [8], [9]]. The TNT approach will therefore likely remain an important platform for the study of novel systemic therapy courses to further improve on current results- both local and distant control- obtained with current doublet and triplet chemotherapy regimens. There are also arguments to be made for continuing with a radiation-first approach when considering the sequencing of the radiation and chemotherapy components in TNT. In the randomized OPRA trial, TME-free survival was improved with the use of radiation (in the form of chemoradiotherapy in this trial) delivered first, although a radiation-first versus chemotherapy-first approach has not been shown to impact overall survival outcomes [7,21].

Nonetheless, there will be a significant subset of patients with LARC who have an incomplete response at the primary treatment site to TNT. Although poor treatment response to neoadjuvant therapy has been associated with an increased risk of distant disease development and poor survival outcomes, surgery is necessary for local control in these circumstances and can still yield a curative outcome for a subset of these patients. It therefore remains an essential consideration in rectal cancer management [1]. In the situation of extended neoadjuvant therapy, the prolonged time interval from radiation to surgery, in particular for radiation-first TNT courses, may be oncologically detrimental because of increased technical difficulties with en bloc mesorectal excision. In recent, updated results from the RAPIDO trial, which enrolled a high-risk group of patients, there was a modest increase in local-regional relapse in the TNT-treated patients relative to the standard arm of chemoradiotherapy without TNT, in the setting of higher rates of incomplete TME specimens, despite higher rates of pathologic complete response. Prolonged time to surgery was also associated with inferior specimen quality in the GRECCAR-6 trial and recent CRONOS analysis [12,13]. Therefore, analysis of further data on the impact of timing from radiation to surgery on surgical outcomes, and local tumor control, is critically important. In our institutional experience with sequential short-course radiation therapy and consolidation chemotherapy, negative surgical margins were obtained in 93 % of surgeries, complete/near-complete TME specimens were obtained in 90 % of surgeries, and optimal surgical outcome (complete/near-complete TME specimen with all surgical margins >1 mm) was achieved in 81 % of patients. These surgical results are generally similar to reported outcomes in other, recent, large series and randomized trials in patients with LARC, including in series with neoadjuvant chemoradiotherapy alone without TNT [4,5,16,[18], [19], [20]]. The high-grade postoperative morbidity rate was also low- comparable to the rates in RAPIDO and PRODIGE 23 as well as those reported in the two arms from ACOSOG Z6051 [4,5,16].

For assessment of intermediate-term local control, we combined the NTx/Surgery and NOM groups as the NTx/Surgery group largely selected for patients not achieving complete response following neoadjuvant therapy, leading to a natural bias. We also used MRI-identified high-risk features to divide patients into two groups- high- and low-risk presentations. The high-risk group shared features similar to the patients enrolled on RAPIDO. Two-year local control was indeed numerically inferior in the high-risk group- 90.4 % versus 97.6 % in the low-risk group, although this difference did not reach statistical significance. Whether improved local control results could be obtained with chemoradiotherapy, in the context of TNT, is unclear. In sum, though, there is no significant signal from our data that extended neoadjuvant therapy with a radiation-first approach compromises surgical specimen quality, postoperative morbidity, or oncologic outcomes (local control).

The main limitation of our analysis is its retrospective design and nature. Patients were treated over a long time span with changes in neoadjuvant management- namely, the duration of consolidation chemotherapy, and the use, or not of postoperative chemotherapy (and its uncertain impact on local control). Nonetheless, the primary endpoint of interest- TME specimen quality- is a well-established outcome in standardized pathology assessment of proctectomy specimens. The range of time interval from completion of radiation to surgery also allowed us to investigate this as a variable of interest for surgical outcomes.

## Conclusion

In conclusion, high rates of optimal TME surgery, with low rates of postoperative morbidity, can be achieved in the context of extended neoadjuvant therapy for locally advanced rectal cancer incorporating short-course radiation therapy. Many patients will achieve complete clinical response and proceed, safely, to nonoperative management, with surgery reserved for salvage. For patients who do proceed to surgery, a significant minority will have achieved a pathologic complete response. For patients with residual disease, the extended interval to surgery after completion of radiation, inherent to any radiation-first TNT approach, does not compromise surgical outcomes nor greatly increase the risk of postoperative morbidity in our experience. Prospective studies and analyses of large series will provide essential data on the value and limitations of TNT in the management of rectal cancer.

## Funding/financial support statement

There was no financial support from any funding agencies for this research.

## Ethical approval statement

This study was approved by our institutional review board (IRB00194005, approval date 12/10/2018).

## CRediT authorship contribution statement

**I-Chia Liu:** Conceptualization, Data curation, Formal analysis, Investigation, Methodology, Software, Supervision, Validation, Visualization, Writing – original draft, Writing – review & editing. **Susan Gearhart:** Data curation, Writing – original draft, Writing – review & editing. **Suqi Ke:** Data curation, Formal analysis, Methodology, Writing – original draft, Writing – review & editing. **Chen Hu:** Data curation, Formal analysis, Methodology, Writing – original draft, Writing – review & editing. **Haniee Chung:** Data curation, Writing – original draft, Writing – review & editing. **Jonathan Efron:** Data curation, Writing – original draft, Writing – review & editing. **Alodia Gabre-Kidan:** Data curation, Writing – original draft, Writing – review & editing. **Peter Najjar:** Data curation, Writing – original draft, Writing – review & editing. **Chady Atallah:** Data curation, Writing – original draft, Writing – review & editing. **Bashar Safar:** Data curation, Writing – original draft, Writing – review & editing. **Eric S. Christenson:** Data curation, Writing – original draft, Writing – review & editing. **Nilofer S. Azad:** Data curation, Writing – original draft, Writing – review & editing. **Valerie Lee:** Data curation, Writing – original draft, Writing – review & editing. **Atif Zaheer:** Data curation, Writing – original draft, Writing – review & editing. **Jacqueline E. Birkness-Gartman:** Data curation, Writing – original draft, Writing – review & editing. **Abhinav V. Reddy:** Data curation, Writing – original draft, Writing – review & editing. **Amol K. Narang:** Data curation, Writing – original draft, Writing – review & editing. **Jeffrey Meyer:** Conceptualization, Data curation, Formal analysis, Investigation, Methodology, Project administration, Supervision, Validation, Visualization, Writing – original draft, Writing – review & editing.

## Declaration of competing interest

Authors ICL, SG, SK, CH, HC, JE, AGK, PAN, CA, BS, VL, AZ, JEBG, AVR, and AKN have no conflicts of interest to declare. ESC reports grants from Haystack, Pfizer, Affirmed, and NextCure and honoraria/speaking fees from Seres Therapeutics. NSA reports receiving institutional funding from Agios, Inc., Array, Atlas, Bayer HealthCare, BMS, Celgene, Debio, Eli Lilly and Company, EMD Serono, Incyte Corporation, Intensity, Merck & Co., Inc. and Taiho Pharmaceuticals Co., Ltd., being a paid consultant for Mirati and QED, and participating on advisory board for Incyte, QED, and Glaxo Smith Kline. JM reports receiving royalty from UpToDate and Springer and sponsored research support from Boston Scientific.

## References

[1] Fokas E., Liersch T., Fietkau R. (2014). Tumor regression grading after preoperative chemoradiotherapy for locally advanced rectal carcinoma revisited: updated results of the CAO/ARO/AIO-94 trial. J Clin Oncol.

[2] Tamburini E., Tassinari D., Ramundo M. (2022). Adjuvant chemotherapy after neoadjuvant chemoradiotherapy and surgery in locally advanced rectal cancer. A systematic review of literature with a meta-analysis of randomized clinical trials. Crit Rev Oncol Hematol.

[3] Ludmir E.B., Palta M., Willett C.G. (2017). Total neoadjuvant therapy for rectal cancer: an emerging option. Cancer.

[4] Bahadoer R.R., Dijkstra E.A., van Etten B. (2021). Short-course radiotherapy followed by chemotherapy before total mesorectal excision (TME) versus preoperative chemoradiotherapy, TME, and optional adjuvant chemotherapy in locally advanced rectal cancer (RAPIDO): a randomized, open-label, phase 3 trial. Lancet Oncol.

[5] Conroy T., Bosset J.F., Etienne P.L. (2021). Neoadjuvant chemotherapy with FOLFIRINOX and preoperative chemoradiotherapy for patients with locally advanced rectal cancer (UNICANCER-PRODIGE 23): a multicenter, randoimsed, open-label, phase 3 trial. Lancet Oncol.

[6] Jing J., Tang Y., Hu C. (2022). Multicenter, randomized, phase III trial of short-term radiotherapy plus chemotherapy versus long-term chemoradiotherapy in locally advanced rectal cancer (STELLAR). J Clin Oncol.

[7] Garcia-Aguilar J., Patil S., Gollub M.J. (2022). Organ preservation in patients with rectal adenocarcinoma treated with total neoadjuvant therapy. J Clin Oncol.

[8] Kim H., Pedersen K., Olsen J.R. (2021). Nonoperative rectal cancer management with short-course radiation followed by chemotherapy: a nonrandomized clinical trial. Clin Colorectal Cancer.

[9] Reddy A.V., Safar B., Jia A.Y. (2022). Nonoperative management following complete response in rectal cancer after short-course radiation therapy and consolidation chemotherapy: clinical outcomes and quality of life measures. Am J Clin Oncol.

[10] Quirke P., Steele R., Monson J. (2009). Effect of the plane of surgery achieved on local recurrence in patients with operable rectal cancer: a prospective study using data from the MRC CR07 and NCIC-CTG CO16 randomised clinical trial. Lancet.

[11] Dijkstra E.A., Nilsson P.J., Hospers G.A.P. (2023). Locoregional failure during and after short-course radiotherapy followed by chemotherapy and surgery compared to long-course chemoradiotherapy and surgery - a five-year follow-up of the RAPIDO trial. Ann Surg.

[12] Lefevre J.H., Mineur L., Kotti S. (2016). Effect of interval (7 or 11 weeks) between neoadjuvant radiochemotherapy and surgery on complete pathologic response in rectal cancer: a multicenter, randomized, controlled trial (GRECCAR-6). J Clin Oncol.

[13] Guzman Y., Rios J., Pareded J. (2023). Time interval between the end of neoadjuvant therapy and elective resection of locally advanced rectal cancer in the CRONOS study. JAMA Surg.

[14] Jia A.Y., Narang A., Safar B. (2019). Sequential short-course radiation therapy and chemotherapy in the neoadjuvant treatment of rectal adenocarcinoma. Radiat Oncol.

[15] Habr-Gama A., Perez R.O., Wynn G. (2010). Complete clinical response after neoadjuvant chemoradiation therapy for distal rectal cancer: characterization of clinical and endoscopic findings for standardization. Dis Colon Rectum.

[16] Fleshman J., Branda M., Sargent D.J. (2015). Effect of laparoscopic-assisted resection vs open resection of stage II or III rectal cancer on pathologic outcomes: the ACOSOG Z6051 randomized clinical trial. JAMA.

[17] Dindo D., Demartines N., Clavien P.A. (2004). Classification of surgical complications: a new proposal with evaluation in a cohort of 6336 patients and results of a survey. Ann Surg.

[18] Angehrn F.V., Schneider R., Wilhelm A. (2022). Robotic versus laparoscopic low anterior resection following neoadjuvant chemoradiation therapy for stage II-III locally advanced rectal cancer: a single-centre cohort study. J Robot Surg.

[19] Schrag D., Shi Q., Weiser M.R. (2023). Preoperative treatment of locally advanced rectal cancer. New Engl J Med.

[20] Hopkins M.B., Geiger T.M., Bethrum A.J. (2020). Comparing pathologic outcomes for robotic versus laparoscopic surgery in rectal cancer resection: a propensity adjusted analysis of 7616 patients. Surg Endosc.

[21] Fokas E., Schlenska-Lange A., Polat B. (2022). Chemoradiotherapy plus induction or consolidation chemotherapy as total neoadjuvant therapy for patients with locally advanced rectal cancer: long-term results of the CAO/ARO/AIO-12 randomized clinical trial. JAMA Oncol.

